# Genotype-Phenotype Correlations in Human Diseases Caused by Mutations of LINC Complex-Associated Genes: A Systematic Review and Meta-Summary

**DOI:** 10.3390/cells11244065

**Published:** 2022-12-15

**Authors:** Emily C. Storey, Heidi R. Fuller

**Affiliations:** 1 School of Pharmacy and Bioengineering, Keele University, Staffordshire ST5 5BG, UK; 2 Wolfson Centre for Inherited Neuromuscular Disease, TORCH Building, RJAH Orthopaedic Hospital, Oswestry SY10 7AG, UK

**Keywords:** LINC complex, nuclear envelope, laminopathies, lamin A/C, *LMNA*, *SYNE1*, *EMD*, nesprin, emerin

## Abstract

Mutations in genes encoding proteins associated with the linker of nucleoskeleton and cytoskeleton (LINC) complex within the nuclear envelope cause different diseases with varying phenotypes including skeletal muscle, cardiac, metabolic, or nervous system pathologies. There is some understanding of the structure of LINC complex-associated proteins and how they interact, but it is unclear how mutations in genes encoding them can cause the same disease, and different diseases with different phenotypes. Here, published mutations in LINC complex-associated proteins were systematically reviewed and analyzed to ascertain whether patterns exist between the genetic sequence variants and clinical phenotypes. This revealed *LMNA* is the only LINC complex-associated gene in which mutations commonly cause distinct conditions, and there are no clear genotype-phenotype correlations. Clusters of *LMNA* variants causing striated muscle disease are located in exons 1 and 6, and metabolic disease-associated *LMNA* variants are frequently found in the tail of lamin A/C. Additionally, exon 6 of the emerin gene, *EMD*, may be a mutation “hot-spot”, and diseases related to *SYNE1*, encoding nesprin-1, are most often caused by nonsense type mutations. These results provide insight into the diverse roles of LINC-complex proteins in human disease and provide direction for future gene-targeted therapy development.

## 1. Introduction

**Figure 1:**
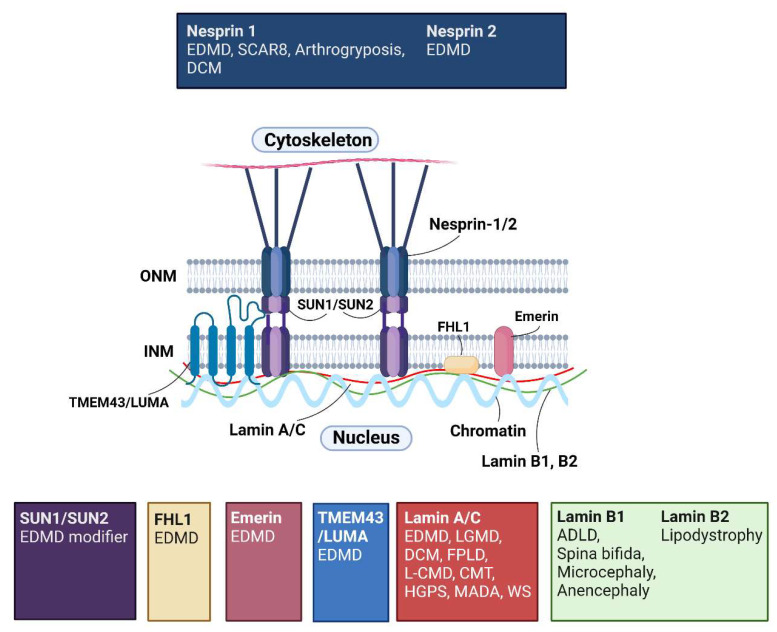
The Linker of Nucleoskeleton and Cytoskeleton (LINC) complex and associated human diseases. The Linker of Nucleoskeleton and Cytoskeleton (LINC) complex primarily consists of SUN1/SUN2 and nesprin proteins, that are located at the inner nuclear membrane (INM) and outer nuclear membrane (ONM), respectively. The respective proteins interact via their Sad1-UNC-84 and Klarsicht/Anc-1/Syne homology (KASH) domains in the lumen of the NE, creating a physical connection between the nuclear lamina (NL) and the cytoskeleton. The NL comprises of type-V intermediate filament proteins, the nuclear lamins. Lamins A/C are A-type nuclear lamins, whilst lamin B1/B2 are B-type lamins. Various other NE proteins interact with the LINC complex including integral INM protein emerin, TMEM43/LUMA and isoforms of four-and-a-half LIM domain protein 1 (FHL1). Mutations in the genes encoding these proteins have been associated with a range of diseases exhibiting different phenotypes. Abbreviations: Emery-Dreifuss muscular dystrophy (EDMD), cerebellar ataxia (SCAR8), dilated cardiomyopathy (DCM), limb-girdle muscular dystrophy (LGMD), familial partial lipodystrophy (FPLD), congenital muscular dystrophy (L-CMD), Charcot-Marie-Tooth disease (CMT), Hutchinson-Gilford progeria syndrome (HGPS), mandibuloacral dysplasia (MADA), atypical Werner syndrome (WS). Created with BioRender.com.

Mutations in genes encoding nuclear envelope (NE) proteins have been associated with a range of human disorders that are collectively known as “nuclear envelopathies” [1,2,3], additionally, the multiple diseases caused by mutations in the nuclear lamins are termed “laminopathies” [4,5]. Mutations in these genes give rise to diseases affecting skeletal and cardiac muscle (such as Emery-Dreifuss muscular dystrophy (EDMD), limb-girdle muscular dystrophy (LGMD), congenital muscular dystrophy (L-CMD) and dilated cardiomyopathy with conduction defects (DCM)), nervous system diseases (including Charcot-Marie tooth disease (CMT), cerebellar ataxia (SCAR8) and amyotrophic lateral sclerosis (ALS)), and metabolic diseases (such as lipodystrophies), amongst others, as shown in (Figure 1). 

The NE is a lipid bilayer structure, functioning to separate the nuclear contents from the cytoplasm. It consists of two lipid bilayer membranes, the inner nuclear membrane (INM) and the outer nuclear membrane (ONM), which are separated by a gap known as the perinuclear space (PNS) [6]. The ONM is a continuation of the rough endoplasmic reticulum, whilst the INM interacts with chromatin and the nuclear lamina (NL), a protein meshwork that composes the nuclear skeleton [7]. Type V-intermediate filament proteins, nuclear lamins, are the major components of the NL [8,9,10]. Lamins A and C are A-type lamins and are the alternatively spliced products of the *LMNA* gene. B-type lamins, lamin B1 and lamin B2, on the other hand, are encoded by two different genes, *LMNB1* and *LMNB2* [11]. The NL is connected to the cytoskeleton through a network called the Linker of Nucleoskeleton and Cytoskeleton (LINC) complex [12]. The central components of the LINC complex are SUN1/SUN2, encoded by *SUN1/SUN2,* and nesprin proteins, derived from *SYNE1/SYNE2* [13]. These proteins are situated in the INM and ONM, respectively [13]. The Sad1 and UNC-84 (SUN) and Klarsicht/Anc-1/Syne Homology (KASH) domains of the respective proteins interact in the PNS to form a connection that spans the INM, PNS and ONM [14,15]. SUN proteins are anchored to the NL by their N-termini, attaching the LINC complex to the NE, whilst the cytoplasmic domains of the nesprins connect to the cytoskeleton [16,17,18]. Additionally, a number of other NE proteins are known to be associated with the LINC complex including INM proteins emerin and TMEM43 (also known as LUMA), and isoforms of four-and-a-half lim domain protein 1 (FHL1) which shuttle between the nucleus and the cytoplasm [19]. 

Though there is some understanding of the structure of LINC complex-associated proteins and how they interact with one another and neighbouring NE proteins [20], it is unclear how mutations in genes encoding them can simultaneously cause the same disease, and different diseases with entirely different phenotypes. Mutations in *LMNA, EMD, SYNE1/SYNE2* and *TMEM43* for example, cause EDMD [21,22,23,24,25], while mutations in *LMNA* affecting the ubiquitously expressed lamin A/C protein lead to more than 15 distinct tissue-specific diseases (Figure 1) [26]. Two hypotheses attempt to explain how one protein could cause entirely different diseases: the structural hypothesis and the gene expression hypothesis. The structural hypothesis proposes that a mutated lamina may result in the nucleus being unable to resist high mechanical strain within cells, particularly within tissues exposed to high mechanical strain [27]. The gene expression hypothesis suggests that *LMNA* mutations affect the regulation of gene expression [11,28]. Accumulating evidence suggests these two may not be mutually exclusive. Further complicating the situation, identical mutations in *LMNA* have been found to cause different conditions [29,30]. While there has been intensive investigation into laminopathies, it is still largely unknown why mutations in the same protein, and especially, why identical mutations can cause different diseases. This hugely complicates the development of therapies for *LMNA*-related diseases.

Whilst research attempting to provide insight into laminopathies has been thorough, studies surrounding the development of diseases associated with mutations affecting other LINC complex-associated proteins and their relation to human diseases is mostly dispersed across many articles and has not been recently synthesised. The aim of this study was to conduct a systematic review into published mutations of genes encoding NE proteins associated with the LINC complex: *LMNA*, *LMNB1*, *LMNB2*, *EMD*, *TMEM43, SUN1/SUN2* and *SYNE1/SYNE2*, and to analyse the list of mutations to determine whether any patterns exist between genetic sequence variants and clinical aspects of the disease. 

## 2. Methods

### 2.1. Overview of Methods

This systematic review of mutations in NE proteins associated with the LINC complex was conducted following Preferred Reporting Items for Systematic Reviews and Meta-Analyses (PRISMA) guidelines [31]. This guidance was developed to ensure the transparent and complete reporting of systematic reviews and meta-analyses. 

### 2.2. Eligibility Criteria

#### Inclusion Criteria

Articles were selected if they clearly described a human mutation in a gene encoding a NE protein associated with the LINC complex (*LMNA*, *LMNB1*, *LMNB2*, *EMD*, *TMEM43*, *SUN1/SUN2, SYNE1/SYNE2*) using the standard nomenclature for describing genetic sequence variants (i.e., describing the nucleotide base pair change and/or amino acid change). Cases where there were multiple mutations in the same gene were included. As identical mutations affecting certain proteins, notably lamin A, are known to cause entirely different diseases, multiple reports of the same mutation were included so that the disease phenotype in different cases could be recorded and compared. Both single and multiple case reports were included.

### 2.3. Exclusion Criteria

Articles were excluded if they did not report a mutation in a gene encoding a NE protein associated with the LINC complex, along with articles that mentioned relevant NE proteins in the title or abstract but failed to describe a mutation in the main body of the article. Cases where more than one mutation was reported in different genes were not included, as it is unclear which is the causative mutation. Asymptomatic carriers were excluded as they do not reflect the patient population that are affected by disease and do not fall within the scope of this study. Non-human or man-made mutations that were created in a laboratory setting were also excluded. It is known that the accumulation of pre-lamin A induces premature senescence within cells, and this is attributed to the development of the following premature aging disorders [32]: Hutchinson-Guilford progeria syndrome (HGPS), atypical Werner syndrome/atypical progeria, mandibuloacral dysplasia and restrictive dermopathy. These diseases were excluded as they are caused by the expression of truncated forms of pre-lamin A that remain farnesylated within cells due to specific mutations that are known to result in the deletion of the ZMPSTE24 protease site [33,34,35]. Additionally, for the same reasons, articles were also excluded if they only mentioned mutations in pre-lamin A. Adult onset autosomal dominant leukodystrophy (ADLD) was excluded as it is known that this disease is caused by a genomic deletion upstream of the lamin B1 gene causing overexpression of *LMNB1*. The causation of this disease is entirely different to other nuclear envelopathies as ADLD is caused by genomic copy number variants as opposed to point or frameshift mutations. Articles were not included if they were not published in English. 

### 2.4. Information Sources

Only bibliographic databases that were deemed relevant to the systematic review topic were searched including Medline (EBSCO) (18th May 2022), CINAHPlus (EBSCO) (18th May 2022) and AMED (EBSCO) (18th May 2022). UniProt, neXtProt and The Human Protein Atlas were accessed as knowledge sources to aid with the analysis of the results yielded in this review.

### 2.5. Search Strategy

No limits or restrictions on date or time-period were applied to the search strategy, however searches were restricted to only include articles that were published in English. No published search filters were used. The database searching yielded a total of 2218 results. There were 875 duplications that were removed using Mendeley Reference Manager and manually, leaving a total of 1343 articles to be screened. The search strategy, including search terms and a full breakdown of search results, can be viewed in detail in Supplementary Materials S1; Tables S1–S3.

### 2.6. Selection Process

Two reviewers blind screened the articles gathered from the searches using the eligibility criteria outlined above. The web-based application Rayyan was used for this process [36]. Any conflicts were recorded and discussed further between the two reviewers until a consensus was reached. 

### 2.7. Data Collection Process

One reviewer independently collected data from each report manually. Data was tabulated and recorded using Microsoft Excel. For each NE protein, mutations were recorded using HGVS cDNA nomenclature and protein nomenclature. In some instances, if this information was not available (i.e., cDNA nomenclature was stated, but protein nomenclature was not or vice-versa), this was manually determined where possible. The type of mutation (i.e., missense, nonsense, insertion, deletion, duplication, frameshift) was also noted using HGVS nomenclature. The exon, codon and protein structure where the mutation is located were recorded or determined based on other information. The number of reports associated with each mutation were recorded if this information was stated in the article (this was therefore recorded as “minimum number of reports of mutation” as this was not always available). Diseases reported as being associated with each mutation were documented and if clinical details were available, disease severity was ranked (either mild, moderate or severe). The raw data from the data collection process can be viewed in Supplementary Materials S2; Tables S4–S6.

### 2.8. Risk of Bias and Certainty Assessment

Articles were rejected in the screening process if the reviewers determined that the information on the mutation was questionable, recorded incorrectly or unclearly. Examples include those where the mutation was not reported using standard nomenclature, or if the position of the mutation on the codon did not align with the cDNA position. Due to the nature of this systematic review, it was possible to manually check whether mutations were correctly recorded by looking at the mutation (the nucleotide or protein change) on the FASTA genetic sequence available on The National Center for Biotechnology Information database.

### 2.9. Limitations of the Study

Some mutations are reported directly to genetic databases and as a result are not published in journal articles. This may be a limitation to this study, as a number of variants may not have been recorded. There are, however, also limitations to database searching. Therefore, we believe that a systematic review was the most appropriate method to use to conduct this study.

## 3. Results

### 3.1. Study Selection

**Figure 2:**
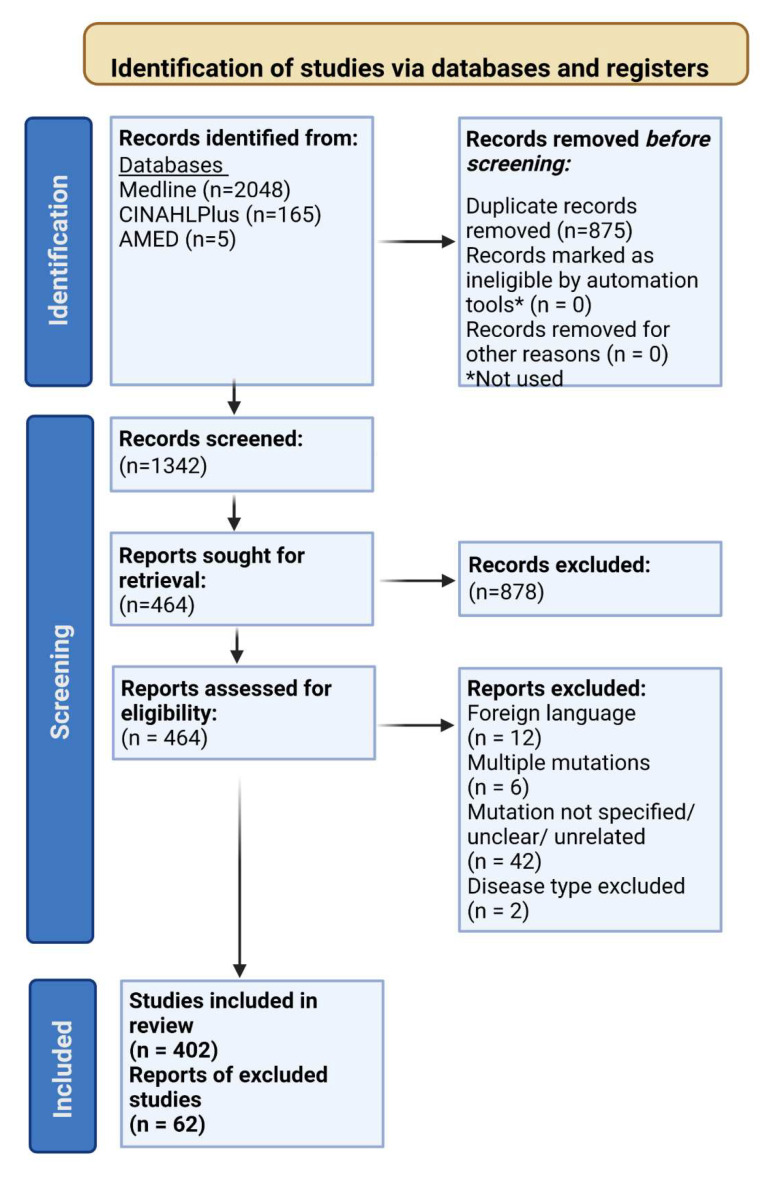
PRISMA flow diagram outlining the search and selection process of identifying records for the systematic review. The databases that were searched were Medline (*n* = 2048), CINAHPlus (*n* = 165) and AMED (*n* = 5), yielding a total of 2218 records. Duplicate records (*n* = 875) were removed prior to screening, then 1254 records were subjected to blind screening by two independent reviewers. 878 reports were excluded, and 464 records were retrieved. This left a total of 464 reports that were assessed for eligibility. There were 402 studies that were included in the review, whilst 62 were excluded. Reports were excluded for the following reasons: foreign language (*n* = 12), multiple mutations (*n* = 6), mutations were not specified, unclear or unrelated (*n* = 42) or contained a disease type that was listed within the exclusion criteria (*n* = 2). Created with BioRender.com.

Bibliographic databases Medline (*n* = 2048), CINAHLPlus (*n* = 165) and AMED (*n* = 5) were searched, yielding a total of 2218 records. Before screening, duplicate records (*n* = 875) were removed. The remaining 1342 records were subjected to blind screening by two reviewers, based on the inclusion and exclusion criteria previously outlined. There were 878 records that were excluded during the first round of screening, this left 464 records that were sought for retrieval. In the final round of screening, 464 reports were assessed for eligibility. Records were excluded due to one of the following reasons: the article was in a foreign language (*n* = 12), multiple mutations in different proteins were described as causing the same disease in the report (*n* = 6), the mutation was not specified, was unrelated to the study or unclear (*n* = 42), the disease type was excluded as stated in the exclusion criteria (*n* = 2). As a result, 402 records were included in the review (Figure 2).

### 3.2. Nuclear Lamins

A high density of *LMNA* mutations at exons 1 and 6 cause striated muscle disease, whilst mutations causing metabolic disease increase in frequency from exon 7 onwards

In total, 519 unique mutations in *LMNA*, the gene that encodes the alternatively spliced products, lamins A and C, were identified. Four mutations were found to be lamin C specific. Lamin C is derived from *LMNA* using an alternative splice site located in intron 10, therefore the C-terminus of lamin C differs to that of lamin A [37].

**Figure 3:**
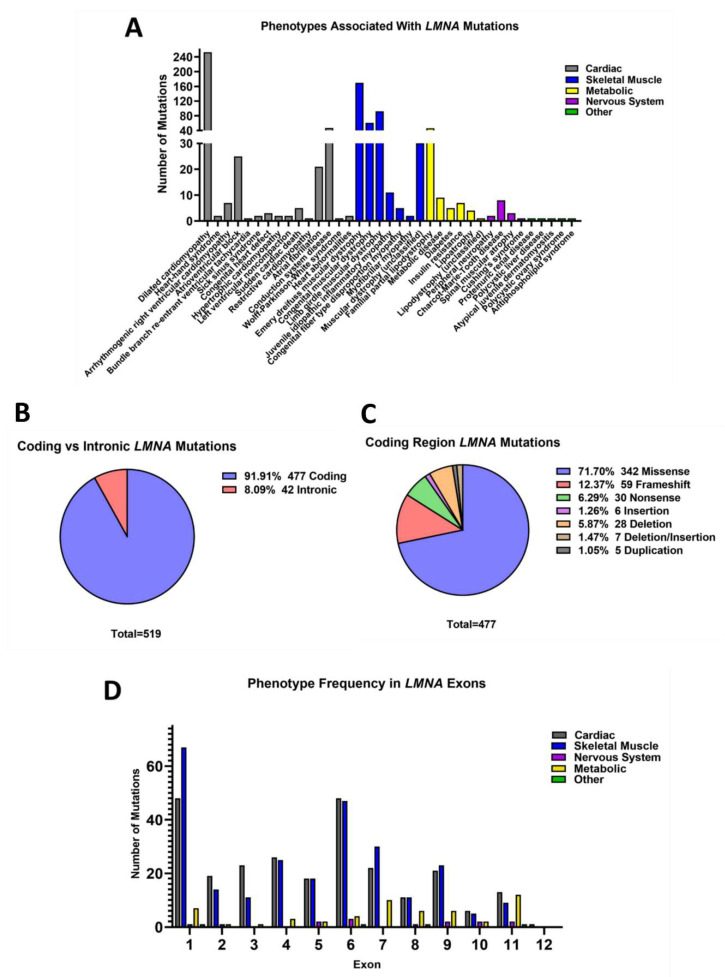
*LMNA* mutations. (**A**) Phenotypes associated with *LMNA* mutations. There were 37 different conditions that were found to be associated with *LMNA* mutations, some which were caused by identical *LMNA* variants. These can be broadly classified into diseases affecting cardiac muscle, skeletal muscle, the metabolic system and the nervous system. A few variants caused diseases that fell into other categories. (**B**) Coding vs. intronic mutations. A total of 519 *LMNA* mutations were identified. 477 (91.91%) were coding mutations, whilst 42 (8.09%) were splice-site variants. (**C**) Coding region mutations. Missense mutations were the most frequently occurring type of *LMNA* mutations (342, 71.70%). Frameshift (59, 12.37%), nonsense (30, 6.29%), deletion (28, 5.87%), deletion/insertion (7, 1.47%), insertion (6, 1.26%) and duplication (5, 1.05%) mutations were also identified. (**D**) Mutations associated with different disease types and their frequency in *LMNA* exons. The most commonly occurring disease types arising from *LMNA* mutations were diseases affecting skeletal (262) and cardiac (260) muscle. These disease types clustered at exons 1 and 6. A total of 55 mutations were associated with metabolic disease, and these mostly appeared to increase in frequency from exon 7 onwards. Only 16 mutations caused nervous system disease and four caused other conditions which fell into different disease categories. Created with GraphPad Prism.

While mutations in *LMNA* have already been associated with more than 15 different phenotypes [26], this study revealed 37 conditions related to *LMNA* variants (Figure 3A). Moreover, identical mutations were found to give rise to different conditions. An astonishing 201 mutations were associated with more than one phenotype, and 99 mutations were linked to disorders affecting entirely different tissues and/or organ systems. Notorious examples of this include the variants p.Arg644Cys, p.Arg453Trp, p.Arg527Pro and p.Ser573Leu which were associated with 11, 6, 5 and 5 different diseases, respectively, each affecting multiple tissues (Table 1). One of the widest ranging examples among these is the p.Arg644Cys variant which has been linked to EDMD, LGMD, L-CMD, atrioventricular block (AVB), DCM, arrhythmogenic right ventricular cardiomyopathy, left ventricular noncompaction, insulin resistance, FPLD, and CMT [30,38,39,40,41,42,43].

There were 477 mutations located in the coding region of the *LMNA* gene (91.92%), and 42 intronic splice-site mutations (8.09%) (Figure 3B). The most frequently occurring type of mutations were missense mutations (*n* = 342), accounting for 71.70% of the mutations found in the coding region of *LMNA.* Frameshift (*n* = 59, 12.37), nonsense (*n* = 30, 6.29%), deletion (28, 5.87%), deletion/insertion (*n* = 7, 1.47%), insertion (*n* = 6, 1.26%) and duplication (*n* = 5, 1.05%) mutations were also identified (Figure 3C). Arginine residues were found to be mutated more often than other amino acids, with 24.32% (*n* = 116) of coding region mutations being the result of arginine mutations. Mutations were distributed across all 12 exons of the *LMNA* gene. The most mutations were found to be located on exon 1, (*n* = 105, 22.01%) and exon 6 (*n* = 75, 15.72%). Very few mutations (*n* = 8) were found within exons 12 and 10. Only one (0.22%) mutation was found to be situated in exon 12, and seven mutations were found in exon 10 (1.51%). 

Given that *LMNA* mutations cause a broad range of diseases, analysis was next performed to determine whether genotype influenced disease phenotype. Using MeSH terms, each laminopathy identified in this study was classified into one of the following disease categories; skeletal muscle disease, cardiac disease, metabolic disease, nervous system disease or other. Each individual *LMNA* mutation was then assigned a disease category, based on the disease(s) that it was found to cause. If a mutation caused more than one disease type, it was counted in more than one category (for example, if a mutation caused DCM and EDMD, it would be categorised as causing skeletal muscle disease and cardiac disease). Disease phenotype was then examined in relation to the position of the causative mutation on the *LMNA* gene, and consequently the affected protein structures and domains. 

**Figure 4:**
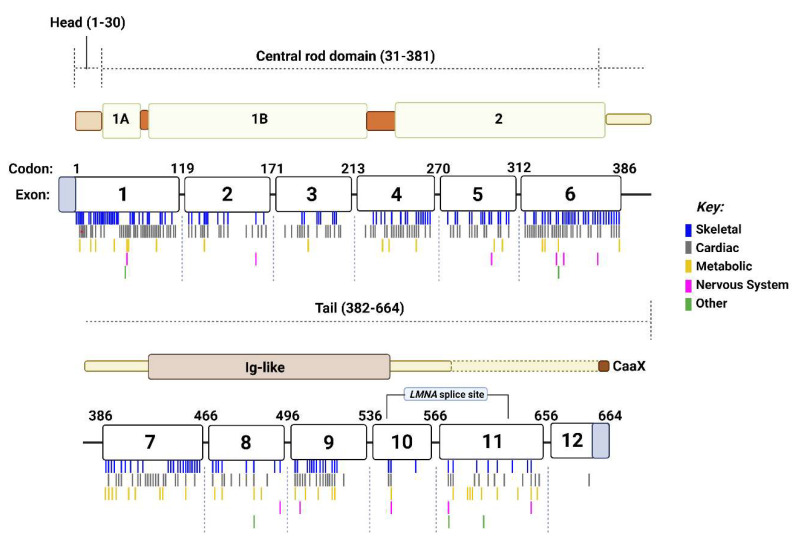
The structure of the *LMNA* gene and protein product lamin A. *LMNA* encodes lamin A/C. Lamin C is produced via a splice site located at intron 10 and therefore it’s C-terminus differs to lamin A. *LMNA* consists of 12 exons. Each coding region *LMNA* mutation was assigned to a disease category based on the disease type it caused. If it caused more than one type of disease, it was considered in multiple categories. These mutations were then marked below the *LMNA* gene at the appropriate codon, in a colour that corresponds to its related disease type(s). Above the *LMNA* gene is the structure of lamin A, consisting of a head, central rod and tail domain. Created with BioRender.com.

The most frequently occurring disease categories associated with *LMNA* mutations were skeletal muscle diseases (*n* = 262 mutations) and cardiac diseases (*n* = 260 mutations). These two disease types seemed to occur most often in exons 1 and 6 (Figure 3D). It was found that 67 mutations causing skeletal muscle disease were in exon 1, and 47 were found to be within exon 6, whilst 48 cardiac disease-associated mutations were located in exon 1, and exon 6 also harboured 48 variants (Figure 3D and Figure 4). Interestingly, in cases where a single mutation caused multiple disease types, most commonly cardiac and skeletal muscle diseases were found to be caused by the same mutation. It is important to also consider that skeletal muscle diseases including EDMD, L-CMD and LGMD do also often cause cardiac abnormalities, therefore there may be some crossover between skeletal and cardiac disease phenotypes. Of 99 mutations known to cause diseases affecting multiple tissues or organ systems, 63 (64.29%) were found to cause both cardiac and skeletal muscle disease, whilst 11 (11.22%) mutations caused cardiac and skeletal muscle disease along with another type of disease(s). There were 55 mutations associated with diseases of the metabolic system. These mutations were distributed throughout the 12 exons of the *LMNA* gene (Figure 3D and Figure 4). The frequency of metabolic disease mutations, however, generally appears to increase from exon 7 onwards (besides the mutations found in exon 1). Exon 7 through to 12 encode the tail region of lamin A (Figure 4). Only 16 mutations were found to cause nervous system disease, whilst 4 mutations caused other conditions which fell into other disease categories.

### 3.3. Fewer Mutations Are Reported in B-Type Lamins Compared to Lamin A/C

Despite their relatively similar size, very few mutations were found in B-type lamins compared to lamins A and C. There were 13 mutations associated with *LMNB1*, all causing neural tube defects (NTDs) including microcephaly (*n* = 5), spina bifida (*n* = 7) and anencephaly (*n* = 1). Only 4 mutations were identified in *LMNB2*, and these were associated with acquired partial lipodystrophy.

### 3.4. Inner Nuclear Membrane

#### 3.4.1. EMD Is Implicated in Diseases Other Than Emery-Dreifuss Muscular Dystrophy

**Figure 5:**
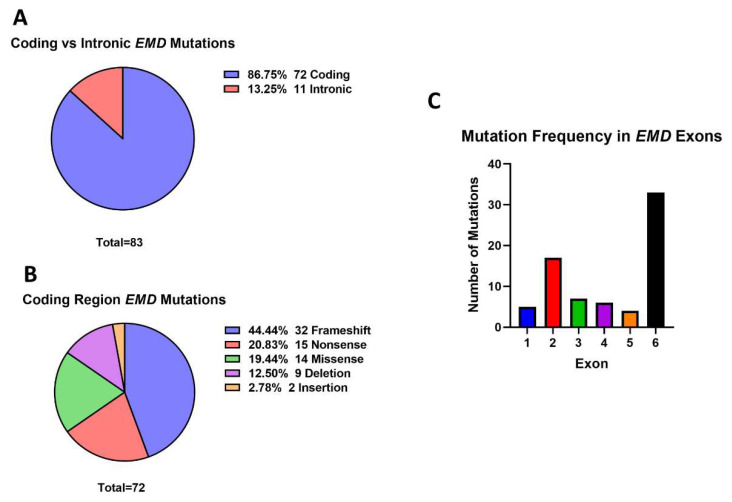
*EMD* mutations. (**A**) Coding vs. intronic mutations. A total of 83 *EMD* mutations were identified. 73 (86.75%) were coding region mutations and eleven (13.25%) were intronic variants. (**B**) Coding region mutations. The largest percentage of coding region mutations were frameshift (32, 44.44%), deletion (9, 12.50%) and nonsense (15, 20.83%) variants. Missense mutations (14, 19.44%) also accounted for a considerable proportion of the mutations identified. A couple of insertion (2, 2.78%) mutations were also identified. (**C**) Mutation frequency in *EMD* exons. Mutations most frequently occurred within exon 6, accounting for 45.21% (33) of total coding region mutations. Exon 2 contained the second highest proportion of mutations (17, 23.29%), followed by exons 3 (7, 9.59%) and 4 (6, 8.22%). Exon 1 and 5 contained the fewest *EMD* variants (5, 6.85% and 4, 5.48%, respectively). Created with GraphPad Prism.

There were 83 mutations found in *EMD*, the gene encoding emerin. We found that 92.86% (*n* = 78) of *EMD* mutations were attributed to EDMD. This study also found that mutations in emerin have been linked to the development of DCM, cardiac conduction disease (CCD), atrial fibrillation (AF), LGMD and rigid spine syndrome (RSS). It is important to note that DCM, CCD and AF are often manifestations of EDMD, although in these cases they have been recorded as isolated diseases, not symptoms of EDMD. Only eleven *EMD* mutations were splice site variants, the remaining 72 (86.25%) mutations were found in the coding region (Figure 5A). Within the coding region of the gene, many mutations were frameshift (*n* = 32, 44.44%), deletion (*n* = 9, 12.50%) and nonsense (*n* = 15, 20.83%) variants. A notable proportion of missense mutations (*n* = 14, 19.44%) were also identified (Figure 5B). *EMD* consists of 6 exons, and mutations were found throughout the emerin gene. However, 39.75% (*n* = 33) mutations were found in exon 6, suggesting that this region may be a mutation hot-spot (Figure 5C). 

#### 3.4.2. Mutations in Inner Nuclear Membrane Protein, TMEM43, and Integral LINC Complex Components SUN1/SUN2, Are Also Linked to Emery-Dreifuss Muscular Dystrophy

In this study, mutations in the INM protein TMEM43 were also found to be linked to the development of EDMD. Only two mutations in *TMEM43* were identified in the literature, however the evidence linking the mutations to EDMD is weak. No other diseases were found to be associated with mutations in this gene. Although we found no mutations in *SUN1/SUN2* that were directly found to cause disease, *SUN1/SUN2* variants have been identified as disease severity modifiers in EDMD when mutations in *LMNA* or *EMD* are present [75].

### 3.5. Outer Nuclear Membrane

**Figure 6:**
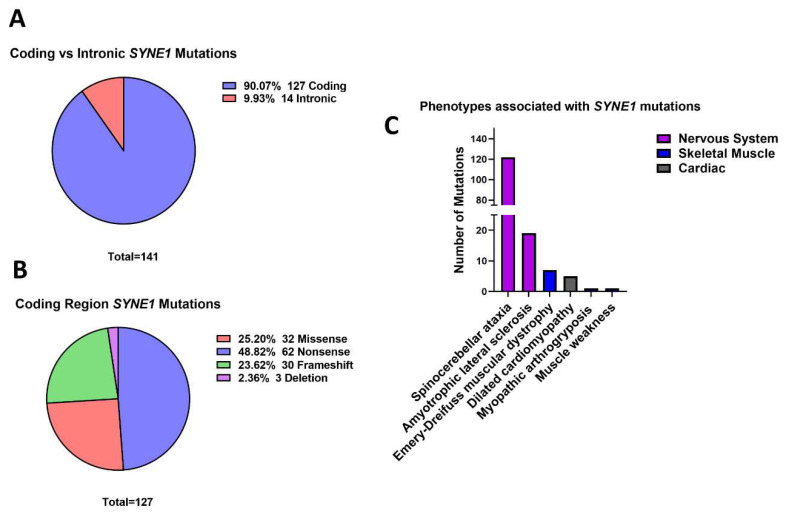
*SYNE1* mutations. (**A**) Coding vs. intronic mutations. There were 127 (90.07%) mutations identified in the coding region of *SYNE1* and 14 (9.93%) were intronic. (**B**) Coding region mutations. Almost half (48.82%, 62) of coding region mutations were nonsense. Many mutations were also missense (32, 25.20%) and frameshift (30, 23.62%) variants. Three (2.36%) deletion mutations were also identified. (**C**) Diseases associated with *SYNE1* variants. The most common disease resulting from *SYNE1* variants was autosomal recessive spinocerebellar ataxia (SCAR8). 122 (85.82%) mutations were related to this disease. Amyotrophic lateral sclerosis was also found to be frequently caused by *SYNE1* mutations (19, 13.48%). Seven cases of Emery-Dreifuss muscular dystrophy (EDMD) were related to *SYNE1* variants, and a handful of mutations were associated with myopathic arthrogryposis (2), dilated cardiomyopathy (DCM) (5) and general muscle weakness (1). Created with GraphPad Prism.

Most often, nesprin-1 nonsense mutations cause nervous system disorders, particularly spinocerebellar ataxia 8 (SCAR8). In total, 141 mutations in *SYNE1* were identified. Out of these, four mutations were found to specifically affect the expression of short isoform nesprin-1α. Only three mutations were identified in *SYNE2.* Most of the *SYNE1* mutations were coding region mutations (*n* = 127, 90.07%), with only 14 intronic mutations being identified (9.93%) (Figure 6A). 48.82% (*n* = 62) of mutations in the coding region of the gene were nonsense (Figure 6B). Additionally, missense (*n* = 32, 25.20%), frameshift (*n* = 30, 23.62%) and deletion (*n* = 3, 2.36%) mutations were identified (Figure 6B). No duplication or insertion mutations were discovered. These mutations were found to be distributed mostly throughout the region of the gene that encodes the spectrin repeats (SRs), with seven mutations being found in the N-terminal calponin homology (CH) domain and three in the KASH domain. 

Mutations in *SYNE1* predominantly cause diseases of the nervous system. Of the mutations identified, 122 were associated with SCAR8, whilst 19 mutations were found to cause ALS (Figure 6C). Fewer mutations were related to the development of EDMD (*n* = 7), myopathic arthrogryposis (*n* = 1), generalized muscle weakness (*n* = 1), and DCM (*n* = 5) (Figure 6C). Two of the *SYNE2* mutations caused EDMD, whilst a single mutation was found to cause ALS.

## 4. Discussion

In this study we systematically reviewed and summarised known mutations in genes encoding LINC complex-associated proteins that are related to human disease, providing a useful resource for researchers in this field. We have demonstrated that there appear to be no obvious correlations between the position of lamin A/C mutations on the *LMNA* gene and different disease phenotypes. However, we found a high frequency of striated muscle disease-causing *LMNA* variants located within exons 1 and 6, whilst mutations associated with metabolic diseases often occurred in exon 7 onwards. A potential emerin mutation “hot-spot” was also identified within exon 6 of *EMD*. We have also observed that most often *SYNE1* nonsense variants lead to the development of nervous system disease. 

*LMNA* variants were associated with 35 different conditions, and in some cases, identical mutations caused diseases with entirely different phenotypes. A notorious example of this is the mutation p.Arg644Cys, which causes 11 different disease phenotypes affecting skeletal and cardiac muscle [38,39,40,41,42], the metabolic system [30], and the nervous system [43]. It remains unclear how this could be, and no answers have arisen from studying the position of known mutations on the *LMNA* gene in relation to different disease phenotypes. This study has, however, revealed that there are no instances in which an identical mutation in the other nuclear envelope genes studied here is responsible for causing so many distinct diseases. One explanation for this could be that other genetic disease modifiers are present in addition to *LMNA* mutations, which may affect the phenotypic outcome of the disease-causing variant. Expressivity modifiers which modulate disease severity have already been identified in conjunction with *LMNA* mutations. Whilst our searches found no mutations in the genes encoding integral LINC complex proteins SUN1/SUN2 were found to directly cause human disease, the presence of *SUN1/SUN2* variants alongside other Emery-Dreifuss muscular dystrophy EDMD-related genes, including *LMNA,* cause a more severe EDMD phenotype [75].The synergistic effects of *LMNA* and *DES* (encoding desmin) or *EMD* mutations have also been found to alter EDMD disease severity [76,77,78]. Perhaps the presence of different modifier genes in combination with *LMNA* mutations could affect lamin A/C expression in variable tissue types leading to the presentation of different diseases. Modifier effects may also influence pleiotropy, causing individuals that share the same target allele to show a range of phenotypes [79,80], though such variation in disease phenotypes resulting from one gene, such as *LMNA,* has never been attributed to modifiers before. *MKS1*, the gene encoding the protein associated with Meckel syndrome type 1, has been identified as a pleiotropic modifier gene in Bardet-Biedl syndrome (BBS). BBS-patients harbouring mutations in both *MKS* and *BBS* (BBS protein) were found to suffer with seizures, which are not typically associated with either MKS or BBS. Hence, the combination of the two mutations generated a completely novel phenotype [81]. Individual modifier genes with pleiotropic effects have also been found in various cystic fibrosis-related phenotypes [82]. 

It is also important to mention that premature aging syndromes including Hutchinson-Guilford progeria syndrome (HGPS), atypical Werner syndrome/atypical progeria, mandibuloacral dysplasia and restrictive dermopathy are also associated with *LMNA* mutations. These diseases are believed to be caused by specific mutations that are known to result in the deletion of the ZMPSTE24 protease site, leading to the accumulation of truncated forms of pre-lamin A that remain farnesylated within cells [33,34,35]. As genotype-phenotype studies have already successfully elucidated that the mutations leading to these diseases affect the ZMPSTE24 site, we did not include this type of diseases within this review. By identifying that *LMNA* mutations related to progeria lead to the deletion of this protease site, it has allowed the advances in our understanding of the development of the disease, which has in turn lead to the identification of potential therapeutic candidates [83]. 

Interestingly, few mutations were identified in B-type lamins, lamin B1 and B2, compared to lamin A/C. This may be because a loss of B-type lamins is incompatible with postnatal life. It has been argued that due to their vital roles in neurodevelopment, B-type lamins are indispensable in mammalian cells. Lamin B1 and B2 deficiency has been found to cause elongated nuclei in neurons and to be essential for neuronal migration in the developing brain [84,85,86]. Moreover, mice harbouring a mutation in lamin B1 did not survive long after birth due to severe developmental abnormalities [87], and lamin B2-deficient mice exhibited brain deformities and died at birth [84]. Another explanation for a lack of mutations in *LMNB1/LMNB2* may be that the expression of A-type lamins compensate when B-type lamins are not present. Some evidence from studying neurodevelopment in mouse models supports this theory, suggesting that low levels or absence of lamin A/C may exacerbate the effects of lamin B deficiency. For example, the absence of *Lmna* in neurons of lamin B1-deficient mouse embryos is associated with abnormal nuclear morphology and severe neuropathology [85]. An alternative hypothesis may be that the loss of one of either lamin B1 or B2 is compensated by the other. A study using *Lmnb1^B2/B2^* and *Lmnb2^B1/B1^* mice (mice that make lamin B2 from the *Lmnb1* locus and vice-versa), however, found that increased production of one B-type lamin doesn’t appear to fully compensate the other, as both mouse models exhibited neuronal abnormalities [88]. Duplications of the *LMNB1* gene have additionally been found to cause autosomal dominant leukodystrophy, although we decided to exclude this due to these mutations being copy number variations [89].

In contrast to the spectrum of diseases caused by *LMNA* mutations, we observed that *EMD* mutations cause multiple diseases, with overlapping clinical phenotypes. Mutations in *EMD* have long been associated with causing EDMD but are also attributed to DCM, AF, CCD and RSS. Whilst these have presented as isolated diseases in certain cases, they are also known symptoms of EDMD [90,91]. A possible explanation as to why *LMNA* mutations cause conditions with very distinct phenotypes, whilst other NE proteins do not, could be due to the highly complex and varied roles of lamin A/C. Besides having structural functions, such as maintaining the integrity of the NE [92,93], lamin A/C has also been implicated in DNA replication, chromatin organisation, cell differentiation, and is known to interact with several transcription factors [94,95,96]. The consequences of mutations on any or all these processes could have a range of effects. Additionally, lamin A/C is known to interact with a number of different proteins that may be expressed in a tissue-specific manner themselves [97,98]. Mutations in *LMNA* may therefore lead to differential interactions of mutant lamins and their associated proteins [99]. Commonly laminopathies are caused by missense mutations, and arginine residues were found to be frequently substituted. Approximately 30% of genetic diseases are due to mutations at arginine and glycine residues [100]. This is a result of arginine being high mutable because of the deamination of 5’-CpG dinucleotides in arginine codons [101], the relatively high frequency of arginine in human proteins, and that arginine mutates to other amino acids with very different chemical properties [100]. Variants affecting arginine residues may have devastating effects on protein stability, as arginine is frequently involved in the formation of salt bridges, and may disrupt and interfere with protein interactions and post-translational modification sites as arginine is also common in protein-active or binding sites [102]. 

Clusters of mutations corresponding to different laminopathies were also found at different exons of the *LMNA* gene. Many mutations causing skeletal myopathies or cardiac diseases occurred within exons 1 and 6. The N-terminal head of lamin A/C is encoded within a region of exon 1, whilst exon 6 corresponds to coil 2 within the central rod domain. These structures are both involved in lamin assembly. Although lamin assembly mechanisms are still not fully understood, lamin dimers are believed to longitudinally associate “head-to-tail” [11,103], whereby the N- and C-terminals of lamin interact. Evidence has also shown that the central rod domains have a high propensity to form “coiled-coil” dimers [104]. Consequently, mutations that affect these structures could impede these processes. Distortions in lamina structure and assembly have been previously observed in cell lines transfected with *LMNA* mutations associated with DCM [105]. Incorrect lamin assembly may then lead to a compromised NL, as previously observed in cells harbouring *LMNA* mutations [106,107,108]. As per the “structural hypothesis” theory that attempts to explain the tissue-specificity of laminopathies, a fragile NL leads to structural weakness resulting in the nucleus being unable to withstand high mechanical stress within cells [109]. This is particularly pertinent within striated muscle, which is exposed to high mechanical tension. 

*LMNA* mutations related to metabolic diseases also appeared to generally increase in frequency from exon 7 onwards, the area which encodes the C-terminal tail region of lamin A. It is understood that the tail domain contains the binding sites for most lamin-binding proteins. Lamin A interacts with and tethers emerin to the NE via a binding site in this region [110,111,112]. This interaction could be altered by mutations, displacing emerin from the NE and as a result, affecting emerin function. Mutations in the tail region of lamin A have previously been shown to affect emerin binding in vivo, however these variants were related to cardiomyopathy and EDMD [113]. Emerin may be involved in metabolic processes [114,115], although the evidence is limited. Lamin A is also thought to regulate 12-lipogenase (12-LOX) through an interaction at a binding site located in the lamin A tail domain [116]. Mounting evidence has shown that 12-LOX contributes to the progression of diabetes, and therapeutic interventions to limit pro-inflammatory 12-LOX metabolites may positively impact on outcomes for diabetes co-morbidities [117,118,119,120]. *LMNA* variants that affect this binding region may result in a dysregulation of 12-LOX which may contribute to diabetes pathology. Other notable binding sites in this region include actin and DNA [95,121,122], but it is unclear how these interactions may be linked to metabolic disease pathology.

As well as lamin A/C mutation clusters being identified within specific exons of *LMNA*, emerin mutations appeared to most often occur within exon 6 of *EMD* suggesting this region of the gene may be a mutation “hot-spot”. It is important to note, however, that exon 6 is the largest exon of the *EMD* gene consisting of 104 amino acids, while exons 4 and 2 are 44 and 35 amino acids in length, and the other exons range from 16–27 amino acids long. While the higher occurrence of mutations in exon 6 may therefore be explained in part because of its larger size, it may not fully account for the differences since exon 4 is 42% of the size of exon 6, but was found to have 6 mutations described, compared to 33 for exon 6. As the majority of these variants are frameshift, deletion and nonsense mutations, they will most likely result in the loss of, or the production of a non-functional truncated form of emerin [25,123,124]. When diagnosing patients with EDMD in resource-restricted settings, it may be useful to consider first sequencing exon 6, given the large number of emerin mutations located in this region. It is important to bear in mind, though, that many *EMD* mutations were located in other exons of the gene.

Most disease-causing *SYNE1* variants result in the production of a truncated form of nesprin-1, because of nonsense mutations. Mutations impairing nesprin-1 function may affect its affinity to interact with both SUN1/SUN2 at the PNS, and with the cytoskeleton in the cytoplasm [12,125]. This would result in the dissolution of the LINC complex, causing the connection between the NL and cytoskeleton to become weakened. *SYNE1* mutations commonly cause SCAR8, suggesting that nesprin-1 is indispensable in the brain. Nesprin-1/2 have been found to be crucial for proper neurodevelopment, and the LINC complex has been found to be the main mediator coupling the nucleus to the cytoskeleton in migrating postmitotic neurons [126]. Furthermore, the LINC complex has been associated with fundamental aspects of embryonic development, particularly radial migration [126]. *SYNE1* variants may hinder these processes resulting in devastating effects. Interestingly *SYNE2* has only been associated with very few cases of human disease, suggesting that maybe nesprin-1 expression might compensate when there is a lack of nesprin-2.

The finding here that almost 50% of *SYNE1* coding region mutations are nonsense suggests that stop-codon readthrough (also known as suppression therapy) should be considered as a treatment option for nesprin-1 related disorders. Suppression therapy utilises pharmacological compounds, which are often aminoglycoside antibiotics, that suppress translation termination at in-frame premature termination codons to restore the translation of a full-length, functional peptide [127,128,129,130,131,132,133]. A number of clinical trials have been conducted where aminoglycoside antibiotics have been administered to patients carrying nonsense mutations with diseases including cystic fibrosis [127,128,134], hemophilia [131] and Hailey-Hailey disease [135] showing varying effects including the partial restoration of full-length, functional protein. Moreover, a nonaminoglycoside known as ataluren (previously PTC124) was the first stop-codon readthrough therapy to gain global approval in 2014 for treatment of nonsense mutation Duchenne muscular dystrophy [136,137]. Stop-codon readthrough may also be applicable for other NE proteins whereby a significant proportion of causative mutations are nonsense.

In this study, we focused on examining how the individual proteins associated with the LINC complex contribute to human disease, but the LINC complex as a whole may also be involved in disease pathophysiology. The LINC complex is involved in rearward nuclear movement and contributes to centrosome orientation. This is facilitated by SUN2 and nesprin-2 assembling into transmembrane-associated nuclear (TAN) lines that link actin cables to the nucleus at the INM through anchorage by emerin and A-type lamins [138]. Depletion of SUN2, nesprin-2, or lamin A/C in C2C12 cells hindered nuclear movement, and in particular, reduction of nesprin-2 also interfered with cell migration and myoblast fusion into myotubes [139]. This suggests that the LINC complex is important for proper myogenic differentiation. The LINC complex is also involved in mechanotransduction, the process by which mechanical stimuli is translated into biochemical signals, allowing cells to adjust their structure and function to the physical environment around them, by allowing force transmission across the NE [140,141]. When exploring nuclear lamin expression in response to external stimuli, it was found that the expression of lamin A/C, relative to B-type lamins, have been found to scale with substrate stiffness both in-vivo and in-vitro [142]. Furthermore, interference of the LINC complex is known to impair intracellular force transmission from the cytoskeleton to the nucleus [143]. When taken together, these findings imply that mutations in any of the LINC complex proteins may also disrupt the LINC complex and its associated functions, which could be related to disease development. From in-vitro studies of cells derived from patients, we also know that mutations in certain LINC complex proteins have implications on others, due to their involvement as components of a protein structure. For example, emerin has been found to be mislocalized in fibroblasts from LGMD patients harbouring *LMNA* mutations, therefore it is important to consider the effect of mutations in LINC complex components on the LINC complex itself [109]. The LINC complex has also been implicated in other disease-related processes, and whilst this was not within the scope of this study, considering the role of these proteins in other biological conditions does allow us to understand more about their function [144]. Mutations in *SYNE1*, for example, have been implicated in human glioblastoma progression and survival [145], and *SYNE1* has also been identified as a potential driver gene in cervical cancer development [146]. Disruption of the LINC complex in glandular epithelial cells was also found to cause the development of aberrant glandular acini, suggesting the LINC complex is important in maintaining tissue architecture, which could be related to cancer pathophysiology [147]. In addition, imbalanced nucleoskeletal connections have been found to create cell polarity defects in HGPS as well as normal physiological aging. As mentioned previously, HGPS is caused by an accumulation of truncated pre-lamin A also known as “progerin” [148]. Progerin affects the mobility of LINC complex-associated proteins, SUN2, nesprin-2 and emerin, which in turn causes defective nuclear positioning and cell polarity which is essential for migration [149].

It is understood that many mutations are reported directly to genetic databases and as a result are not published in journal articles. This may be a limitation to this study and as a result a number of mutations may not be recorded and therefore missing in the final analysis. However, there are also limitations to database searching. We found that it was not possible to easily filter mutations by disease or phenotype, therefore it was more appropriate to conduct a systematic review into the literature where we could find clinical details within journal articles. It is also important to consider that some patients with mutations in genes encoding NE proteins displayed “like-phenotype” symptoms. These may not necessarily be strictly classified as diseases but may be diseases mimicking the phenotypes of other diseases. This is particularly relevant to the many diseases associated with *LMNA* variants. It is necessary to include these phenotypes within this study as it is interesting and valuable to report that these phenotypes are linked to mutations in these genes, even if these cases don’t allow for a full clinical classification. This could be considered a limitation of the literature, and what is reported within the literature. Another constraint is that some variants, such as the *LMNA* p.Arg644Cys mutation, are controversial in that they have been reported as both pathogenic and benign [41,150]. We tried to mitigate against this by ranking the pathogenicity likelihood of variants, but it is possible that some mutations may not be pathogenic. Considering the potential for differences between information contained within genetic databases compared with the literature, it would be useful in future to undertake a systematic comparison of the two information sources. This would allow for a more robust method of identifying discrepancies between the pathogenicity ranking of mutations, and whether results of phenotype-genotype comparison studies would be altered if some variants are re-classified as benign.

## 5. Conclusions

Overall, many unanswered questions remain surrounding *LMNA* mutations, and why faults with this one protein can cause a spectrum of different diseases, especially since this phenomenon is not seen with in other NE proteins. Despite these uncertainties, other observations were made which offer some insight into the potential downstream consequences of lamin A/C mutations. Striated muscle diseases appear to be most frequent in exons 1 and 6 of *LMNA*, which correspond to areas of the protein involved in lamin-lamin interactions. Subsequently, mutations located in these exons may prevent correct lamin assembly. Variants associated with metabolic diseases generally increase in frequency within the area of *LMNA* which encodes the tail domain of lamin A/C. This region facilitates interactions between numerous proteins and lamin A/C. These interactions could be disrupted by mutations that affect these binding sites. Additionally, exon 6 of *EDM* may be an emerin mutation “hot-spot”, which is perhaps useful to know when diagnosing EDMD, while nonsense *SYNE1* mutations are frequently the cause of SCAR8, suggesting a compromised LINC complex particularly disrupts processes in the brain. Stop-codon read-through therapy may be a beneficial treatment option for SCAR8 and more research may be needed in this area. Overall, these results offer insight into the varied roles of LINC-complex associated proteins in human disease and provide direction for future gene-targeted therapy development. Moreover, identifying conditions affected by mutations in different NE proteins may allow for sharing of therapies or therapeutic development strategies. 

## Figures and Tables

**Table 1:** Examples of *LMNA* mutations known to cause distinct phenotypes. The p.Arg644Cys variant was associated with 11 phenotypes affecting skeletal and cardiac muscle, metabolism, and the nervous system. Whilst the p.Arg453Trp, p.Arg527Pro and p.Ser537Leu mutations each caused diseases associated with three different tissue or organ systems. Abbreviations: Emery-Dreifuss muscular dystrophy (EDMD), limb-girdle muscular dystrophy (LGMD), congenital muscular dystrophy (L-CMD), atrioventricular block (AVB), dilated cardiomyopathy (DCM), arrhythmogenic right ventricular cardiomyopathy (ARVC), left ventricular noncompaction (LVNC), insulin resistance (IR), familial partial lipodystrophy (FPLD), Charcot-Marie-Tooth disease (CMT), congenital fibre type disproportion myopathy (CFTD).

Protein Nomenclature	c.DNA Nomenclature	Associated Diseases				Min. Number of Reports of Variant	References
		Skeletal Muscle	Cardiac	Metabolic	Nervous System		
p.Arg644Cys	1930C > T	EDMD, muscular dystrophy, LGMD, L-CMD	AVB, DCM, ARVC, LVNC	IR, FPLD	CMT	23	[24,30,39,40,41,42,43,44,45,46,47]
p.Arg453Trp	1357C > T	EDMD, LGMD, L-CMD, CFTD	DCM	FPLD		37	[44,48,49,50,51,52,53,54,55,56,57,58,59,60,61,62,63,64,65,66,67,68,69]
p.Arg527Pro	1580G > C	EDMD, LGMD, L-CMD	DCM	FPLD		21	[44,46,56,22,58,60,65,66,68,69,70,71]
p.Ser573Leu	1718C > T	LGMD	DCM, AVB	Diabetes, FPLD		14	[44,45,72,73,74]

## Supplementary Materials

The following supporting information can be downloaded at: https://www.mdpi.com/article/10.3390/cells11244065/s1, Supplementary Materials S1 (Excel document containing Tables S1–S3) and Supplementary Materials S2 (Excel document containing Tables S4–S6).
